# Effects of Different pH-Values on the Nanomechanical Surface Properties of PEEK and CFR-PEEK Compared to Dental Resin-Based Materials

**DOI:** 10.3390/ma8084751

**Published:** 2015-07-27

**Authors:** Shuai Gao, Shanshan Gao, Baohua Xu, Haiyang Yu

**Affiliations:** 1 State Key Laboratory of Oral Diseases, West China Hospital of Stomatology, Sichuan University, NO. 14, 3rd Section of Ren Min Nan Road, Chengdu 610041, China; E-Mails: bian_xiaojin@163.com (S.G.); christina12357@163.com (S.G.); 2 China-Japan Friendship Hospital, Yinghua Road, Chaoyang District, Beijing 100020, China; E-Mail: drxubaohua@163.com

**Keywords:** PEEK, carbon fiber-reinforced PEEK, nanoindentation, nanoscratching, nanomechanical properties

## Abstract

The study determines the stability and durability of polyetheretherketone (PEEK) and a carbon fiber-reinforced PEEK (CFR-PEEK) with 30% short carbon fibers, a dental composite based on Bis-GMA and polymethylmethacrylate (PMMA) under the influence of different pH-values of the oral environment *in vitro*. Nanomechanical properties were investigated by nanoindentation and nanoscratch tests before and after incubation of the specimens at 37 °C for 30 days in artificial saliva with pH-values of 3, 7 and 10, respectively. Nanoindentation and nanoscratching tests were performed using the Hysitron TI950 TriboIndenter to evaluate the reduced elastic moduli, nanohardness, viscoelasticity, friction coefficient and residual scratch profiles. After treatment, the nanomechanical properties of unfilled PEEK did not change. The reduced elastic moduli and nanohardness of the carbon fiber-reinforced PEEK increased significantly. The reduced elastic moduli and nanohardness of CHARISMA decreased. The plasticity of all materials except that of the unfilled PEEK increased. This indicates that different pH-values of the artificial saliva solutions had no obvious influences on the nanomechanical properties of the PEEK matrix. Therefore, the aging resistance of the unfilled PEEK was higher than those of other materials. It can be deduced that the PEEK matrix without filler was more stable than with filler in the nanoscale.

## 1. Introduction

Polyetheretherketone (PEEK) is a high-performance semicrystalline thermoplastic polymer with good mechanical properties and excellent biocompatibility [1]. PEEK is also an anti-aging material with excellent chemical and physical stability and can resist radiation and sterilization damage [2]. On account of these characteristics, PEEK has been widely used as bone substitute in the orthopedic field [3,4,5,6]. Recently, PEEK has been introduced into the field of prosthetic dentistry. Many applications such as dental implants, provisional abutments or clamps for removable dental prostheses are described in the literature [7,8,9]. Furthermore, with a reported mean fracture load of 1383 N for three-unit fixed dental prostheses (FDPs), PEEK has also been considered as a suitable material, especially, for FDPs in load-bearing areas [10]. According to a recent research, adequate tensile bond strengths between resin and PEEK could be achieved after surface treatment with sulfuric acid or air-abrasion. It could be demonstrated that the optical disadvantageous properties due to a low translucency and a dark yellow or grayish color of PEEK can be improved by veneering with a dental composite [7,11], making PEEK a potential restorative material in the anterior esthetic prosthetic region of oral cavity.

Long-term durability is one of the most important properties of conventional dental composite materials. The oral cavity is a complex system, which includes saliva components, chewing force, and thermal and chemical dietary, can cause biodegradation of dental composite materials. During this process, the leachable products will be produced and may induce a series of biological responses on cells and tissues [12]. Since PEEK is increasingly used in the field of dentistry, its duration and stability in the oral cavity must be ensured. Previous studies reported that PEEK remains stable in nearly all organic and inorganic chemicals [13,14], and many researches demonstrated that PEEK can persist in many complex aging environments [2]. However, no studies have yet concerned the durability and stability of PEEK in the oral cavity environment, whereas an accurate understanding of the long-term performance of PEEK and CFR-PEEK is essential for their application in the field of dentistry.

The purpose of this study was to evaluate the influence of the exposure to different pH-values of artificial saliva solutions on the surface properties of an unfilled PEEK, a carbon fiber-reinforced PEEK (CFR-PEEK) in comparison with conventional dental resin-based materials by using nanoindentation and nanoscratching tests [15,16].

## 2. Materials and Methods

### 2.1. Materials

A commercially available unfilled PEEK (PEEK 450G, Victrex, UK) and a CFR-PEEK (PEEK 450CA30, Victrex, Thornton Cleveleys, UK), a dental resin-based composite (CHARISMA, HERAEUS, Wehrheim, Germany), and a dental polymer matrix (PMMA, QC-20, DENTSPLY, Tianjin, China) were investigated in this study. The PEEK materials had the same polymer matrix, whereas CFR-PEEK contained 30 wt % carbon fibers. The details of the studied materials are listed in Table 1.

**Table 1:** Manufacturers’ information for the studied materials.

Materials	Organic Matrix	Filler	Content of Filler
PEEK 450G	Polyetheretherketone	None	None
PEEK 450CA30	Polyetheretherketone	Short carbon fiber: 6–9 µm (diameter)	30 wt %
CHARISMA	Bis-GMA(2,2-Bis[4-(2hydroxy-3-methacryloxypropyl-1-oxy) phenyl]pro-pane)	Barium-aluminaborosilicate, Silica: 0.02–0.07 µm	64 wt %
PMMA	Poly(methyl methacrylate)	None	None

Disk specimens of PEEK (*n* = 40) and CFR-PEEK (*n* = 40) were machined into disks of 8 mm in height from an extruded 10 mm diameter rod. The dental resin-based composite CHARISMA (*n* = 40) disk specimens and PMMA (*n* = 40) specimens with a diameter of 10mm and a thickness of 8 mm were prepared following the manufacturers’ recommendations. All the specimens were grinded with silicon carbide abrasive papers (Struers, Copenhagen, Denmark) in a sequence of decreasing abrasiveness (P800, P1200, P2400, and P4000-grit) under continuous water cooling. Specimens were further polished using a sequence of felt cloths (Dac, Struers, Denmark) under periodical lubrication with alumina suspension slurry (Struers, Copenhagen, Denmark) of 3 µm for 5 min and OP-Nondry (Struers, Copenhagen, Denmark) of 0.04 µm for 10 min on a polishing machine (Struers, Copenhagen, Denmark). The average roughness of the resultant surface was less than 5 nm, which evaluated and confirmed by the scanning probe microscope (SPM) equipped in the TriboIndenter system (Hysitron Inc. TI950, Minneapolis, MN, USA). The samples were then ultrasonically cleaned for 5 min in deionized water (KQ-50B, Shumei, Kunshan, China).

The SAGF^®^ medium was chosen as artificial saliva in this work and its composition is given in Table 2 [17,18]. This medium was chosen as treatment solution in order to bring our trials closer to real in-mouth conditions. The pH value of artificial saliva was adjusted to 7.0 with CO_2_. To simulate the variations of the pH values of the oral environment, the artificial saliva were was also adjusted to pH = 3.0 and pH = 10.0 with lactic acid and sodium hydroxide, respectively [19]. The container for the solutions and samples were hermetically sealed to eliminate the influence of CO_2_.

**Table 2:** Composition of the artificial saliva (SAGF medium).

Components	Concentration (mg L^−1^)
NaCl	125.6
KCl	963.9
KSCN	189.2
KH_2_PO_4_	654.5
Urea	200.0
NaSO_4_·10H_2_O	763.2
NH_4_Cl	178.0
CaCl_2_·2H_2_O	227.8
NaHCO_3_	630.8

Materials were randomly divided into four groups (ten samples in each group) based on the exposure conditions (pH value) used for the final treatment as follows: Group 1, pH 3.0 (acidic artificial saliva, adjusted by lactic acid); Group 2, pH 7.0 (pH-neutral artificial saliva); Group 3, pH 10.0 (alkaline artificial saliva, adjusted by sodium hydroxide); and Group 4, control (no exposure to artificial saliva). Specimens of groups 1–3 were individually immersed into 20 mL of each solution and stored at 37 °C for 30 days [18]. The artificial saliva was replaced and checked daily.

### 2.2. Nanoindentation and Nanoscratching

In this research, the surface mechanical properties of the samples were studied by nanoindentation and nanoscratch tests. All works were carried out using the commercially available TriboIndenter system (Hysitron Inc. TI950, Minneapolis, MN, USA) with a Berkovich type indenter tip for the indentation test and a conical diamond indenter tip for the scratch test. The calibration of the triboindenter system was performed on a fused quartz standard specimen. All tests of specimens were undertaken at room temperature and the thermal drift was calculated by the system automatically. The indents were located 30 μm apart to avoid the influence of residual stresses from adjacent impressions [20].

During the test, load and displacement were continuously monitored. Nanomechanical properties such as nanohardness and reduced elastic moduli could be calculated using well-established equations based on the elastic contact theory. The reduced elastic moduli were calculated by the Oliver and Pharr method [21].

To avoid assumptions about contact conditions including the tip shape inherent in some definitions, plasticity index [21] (ψ, defined in terms of energy) was used. The plasticity index was obtained by the ratio of energy irreversibly dissipated to the total energy expended in deforming a material subject to a given external load:

$$\Psi =W_{\mathrm{ir}}/W_{\mathrm{ir}}+W_{r}$$

**Figure 1:**
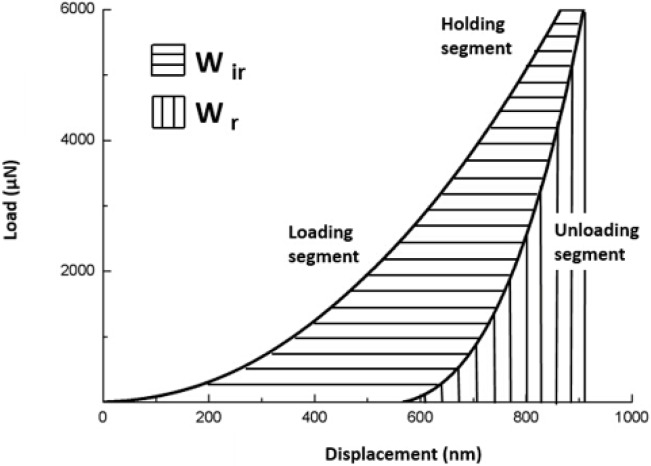
Schematic of load-displacement data recorded during an indentation. The load segment, displacement segment and areas corresponding to the reversible and irreversible work, *W*_r_ and *W*_ir_ respectively, are shown.

In this equation, *W*_ir_ and *W*_r_ represent the irreversible work done during indentation and the reversible work recovered by viscoelastic processes during the unloading stage respectively. In the load-displacement curve, the *W*_ir_ is the area enclosed by the loading and unloading portions and *W*_r_ represents the area underneath the unloading part of the curve (see Figure 1).

#### 2.2.1. Nanoindentation Test Protocol

The indentation tests were performed in the load control mode to obtain the nanohardness, reduced elastic moduli and plasticity index. The loading function of indentation in this work consisted of a 20-s linear loading and 20-s unloading segment together with a holding of 20 s at the peak load, which was used to reduce the influence of creeping effect [20]. The maximum load applied by the indenter to examine the specimens was 6000 μN.

#### 2.2.2. Nanoindentation Creep and Recovery Test Protocol

**Figure 2:**
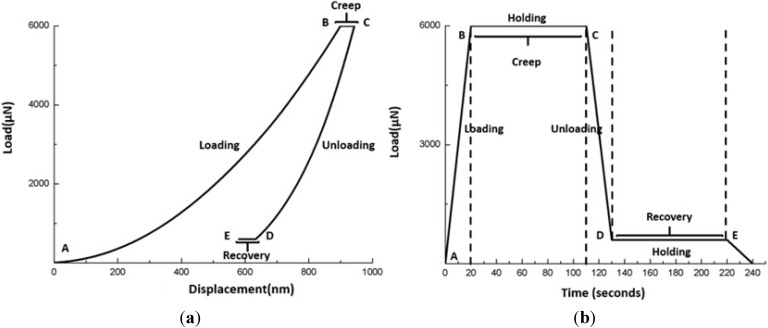
Schematic of nanoindentation creep and recovery test. (**a**) load-displacement curve. (**b**) load-time curve.

This tests were operated in the force-control mode and consisted of four steps (Figure 1): loading (A to B) for 20 s, first holding at a maximum load (6000 μN) for 90 s (B to C), unloading for 20 s (C to D), and the second holding at a minimum load (600 μN) for 90 s (D to E). From the data recorded by the system [22], the nanoindentation creep was calculated through subtracting the displacement value (nm) at point B (Figure 2) from the displacement value (nm) at point C (Figure 2). The result was the “nanoindentation creep”. The difference in displacement at the start and at the end of the second hold period (D–E) was taken as the “nanoindentation recovery”.

The load function used for the indentation creep and recovery test is illustrated schematically in Figure 2.

#### 2.2.3. Nanoscratching Test Protocol

The nanoscratching tests were performed under the normal load of 1000 μN [23], and the length of the scratch was 10 μm with a constant scratching speed of 0.5 μm/s. Each sample was scratched another ten times to achieve reliable results. In a nanoscratching test, the coefficient of friction was determined from the ratio of the lateral force to the normal force.

## 3. Statistical Analysis

A non-parametric test (the Kruskal-Wallis test) was used to analyze the reduced elastic moduli and nanohardness of different regions of CFR-PEEK data. A one-way ANOVA (Dunnett T3 post hoc test) was used to analyze the rest data collected from this study. The significance level established at *p* ≤ 0.05.

## 4. Results

### 4.1. Nanoindentation Results

**Figure 3:**
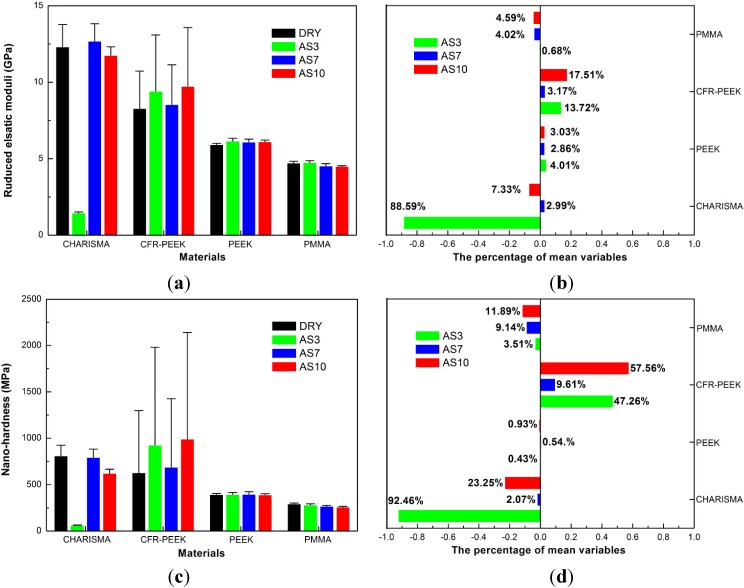
(**a**,**b**) Reduced elastic moduli (GPa) and percentage of mean variables, means of 60 indentations (6 indentations each sample) of CHARISMA, CFR-PEEK, PEEK, and PMMA. The bars represent standard deviations. (**c**,**d**) Nanohardness (MPa) and percentage of mean variables, means of 60 indentations (6 indentations each sample) of CHARISMA, CFR-PEEK, PEEK, and PMMA. The bars represent standard deviations. In (**b**) and (**d**), the negative number on the *X* axis represents decrease, and the positive number represents increase.

The reduced elastic moduli and nanohardness values of CHARISMA, PEEK, CFR-PEEK, and PMMA are plotted in Figure 3. For the reduced elastic moduli and nanohardness of PEEK, no significant differences of nanohardness were observed among these groups (*p* = 0.505). Although the reduced elastic moduli of the control group was significantly different from other groups, little difference was observed among other groups. Furthermore, it is interesting that the reduced elastic moduli and nanohardness of CFR-PEEK treatment groups increased, especially in the CFR-PEEK AS3 (increased 13.72% and 47.26%, respectively) and CFR-PEEK AS10 (increased 17.51% and 57.56%, respectively) groups. Because the size of carbon fiber fillings was bigger than the indenter tip, the test values of different regions in this composite were various, making the standard deviation of CFR-PEEK much bigger.

After storage in different pH value artificial saliva, the reduced elastic moduli and nanohardness of CHARISMA decreased remarkably, especially in the CHARISMA AS3 group (decreased 88.59% and 92.46%, respectively).

Moreover, the nanohardness of PMMA significantly decreased after the storage treatment. The lowest nanohardness test data was in the PMMA AS10 group (decreased 11.89%).

**Figure 4:**
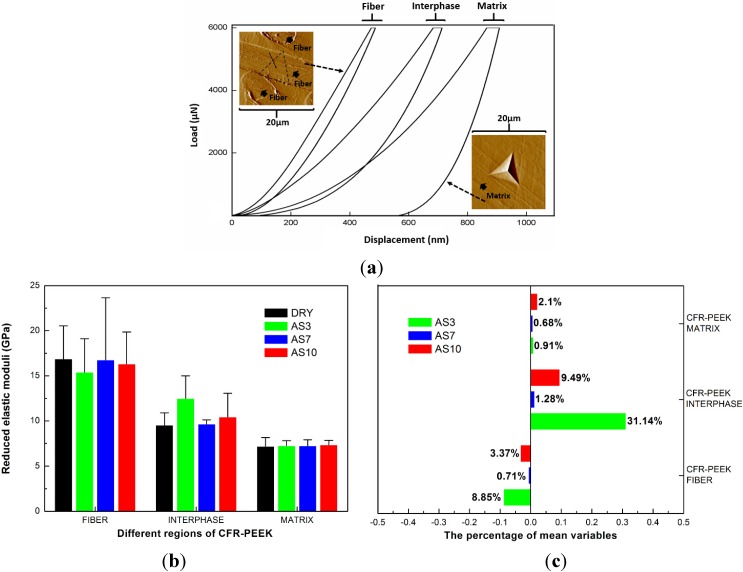

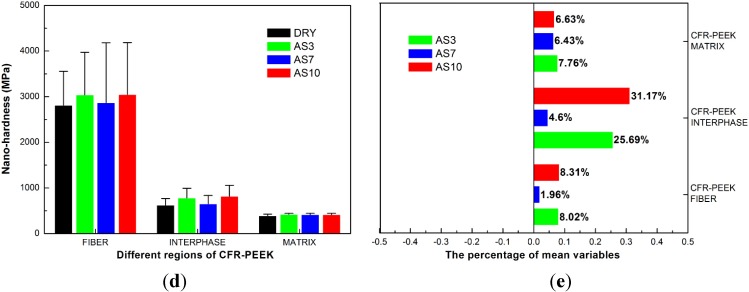
(**a**) Schematic of different load-displacement curves represents different regions of CFR-PEEK. The distinct permanent set profile can be seen in the matrix region after a 6000-μN indentation (left SPM figure). When indentation was performed on a carbon fiber, almost no permanent set was observed. The region pointed by the black thin arrow mark is the position where indentation was performed (right SPM figure). (**b**,**c**) Reduced elastic moduli (GPa) and percentage of mean variables of different regions of CFR-PEEK. The bars represent standard deviations. (**d**,**e**) Nanohardness (MPa) and percentage of mean variables of different regions of CFR-PEEK. The bars represent standard deviations. In the (**b**) and (**d**), the negative number on the X axis represents a decrease and positive number represents an increase.

According to the different load-displacement curve, which can be seen in Figure 4, there were typically three distinct areas that represented the matrix, the carbon fibers, and the interphase respectively [21]. To investigate the variation of CFR-PEEK, the reduced elastic moduli and nanohardness of these regions were analyzed respectively.

It could be seen that the reduced elastic moduli and nanohardness of CFR-PEEK significantly increased in the interphase regions (*p* = 0.002). The test values of interphase regions were much higher than the others in the AS3 groups (increased 31.14% and 25.69%, respectively) and the AS10 groups (increased 9.49% and 31.17%, respectively).

**Figure 5:**
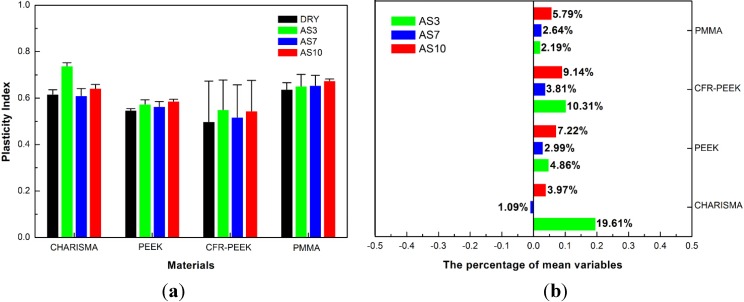
(**a**,**b**) Plasticity index and percentage of variations, means of 60 indentations (6 indentations each sample) of CHARISMA, PMMA, PEEK, and CFR-PEEK. In (**b**), the negative number on the *X* axis represents decrease and positive number represents increase. The bars represent standard deviations.

The plasticity index, which characterizes the viscoelasticity of the materials, is displayed in Figure 5. It could be seen that nearly all the plasticity index increased after treatment, which indicated that all these studied materials performed more permanent deformation and less reversible work during the processes of viscoelastic recover in one indentation. The highest plasticity index of PMMA (increased 5.79%) and PEEK (increased 7.22%) were caused by the treatment of AS10. However, the highest plasticity index of CHARISMA (increased 19.61%) and CFR-PEEK (increased 10.31%) were contributed to the AS3 treatment.

The mean data of the nanoindentation creep, recovery, and percentage of nanoindentation recovery are presented in Table 3. For the nanoindentation creep of PEEK and PMMA, there were no significant differences between the control group and experimental groups. For the nanoindentation recovery of PEEK, no statistical differences were observed among DRY, AS3, and AS7 groups.

The percentage of nanoindentation recovery was the ratio of nanoindentation creep and nanoindentation recovery, which represented the capability of elastic recovery after a long-time indentation. In this study, the percentage of nanoindentation recovery of PEEK, CFR-PEEK, and PMMA decreased after treatment. For PEEK, the highest percentage of nanoindentation recovery could be seen in the PEEK DRY group (1.22). Nevertheless, after AS3 and AS10 treatment, the percentage of nanoindentation recovery of CHARISMA increased.

**Table 3:** Mean value (standard deviations) of the nanoindentation creep and recovery parameters of the treatment groups and the control group. Each nanoindentation creep test value is the mean of 30 indentations (3 indentations each sample).

Materials (Treatment Group)	Nanoindentation Creep (nm)	Nanoindentation Recovery (nm)	Percentage of Nanoindentation Recovery (%)
CHARISMA (DRY)	98.41(21.59) ^a^	55.45(24.08) ^a^	0.56
CHARISMA (AS3)	337.78(14.98) ^b^	233.98(11.87) ^b^	0.69
CHARISMA (AS7)	112.27(35.15) ^a^	58.56(20.14) ^a^	0.52
CHARISMA (AS10)	66.97(11.49) ^c^	57.46(12.62) ^a^	0.86
PEEK (DRY)	64.43(25.88) ^e^	78.36(32.23) ^e^	1.22
PEEK (AS3)	59.23(20.96) ^e^	58.38(31.13) ^e^	0.99
PEEK (AS7)	74.11(27.59) ^e^	72.17(31.65) ^e^	0.97
PEEK (AS10)	53.63(6.56) ^e^	45.89(6.95) ^f^	0.86
CFR-PEEK (DRY)	76.67(20.79) ^i^	92.91(30.61) ^i^	1.21
CFR-PEEK (AS3)	103.34(32.85) ^j^	31.28(17.46) ^j^	0.30
CFR-PEEK (AS7)	52.79(12.4) ^k^	27.24(9.68) ^j^	0.52
CFR-PEEK (AS10)	71.39(18.60) ^i^	23.05(9.76) ^j^	0.32
PMMA (DRY)	153.73(19.39) ^m^	144.54(20.99) ^m^	0.94
PMMA (AS3)	162.35(5.98) ^m^	125.34(7.27) ^n^	0.77
PMMA (AS7)	150.61(4.55) ^m^	122.55(4.52) ^n,o^	0.81
PMMA (AS10)	161.49(7.01) ^m^	128.96(7.18) ^n,p^	0.79

Same superscript letters indicate homogenous subsets (*p* > 0.05).

### 4.2. Nanoscratch Results

**Figure 6:**
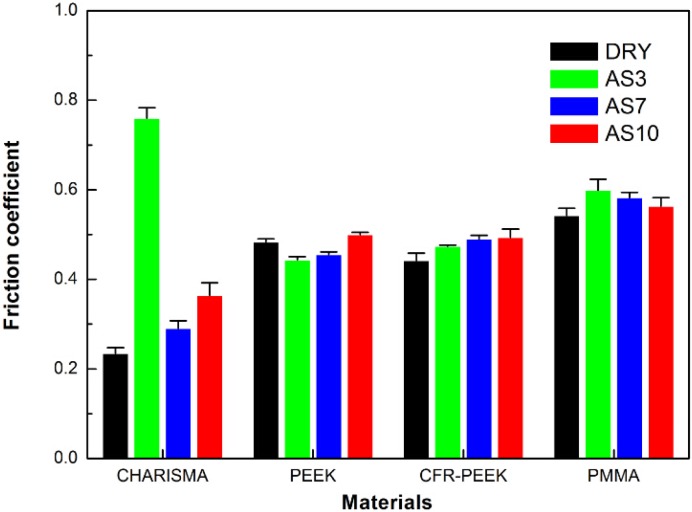
Friction coefficient, means of 30 nanoscratch (3 indentations each sample) of CHARISMA, PMMA, PEEK, and CFR-PEEK before and after treatment. The bars represent standard deviations.

The typical frictional coefficients of all the samples are plotted in Figure 6. The lowest friction coefficient was presented in the CHARISMA DRY group (0.23). However, after treatment, the test value increased to 0.76 significantly. For PEEK and CFR-PEEK, the lowest frictional coefficients could be seen in the AS3 group (0.44 and 0.37), and the highest test values were in the AS10 group (0.50 and 0.49). However, according to the column chart of PEEK and CFR-PEEK, it could be seen that the storage treatment had no obvious influences on them. After storage in the artificial saliva, the friction coefficient of PMMA increased, and the highest value was reported in the PMMA AS3 group (0.59).

**Figure 7:**
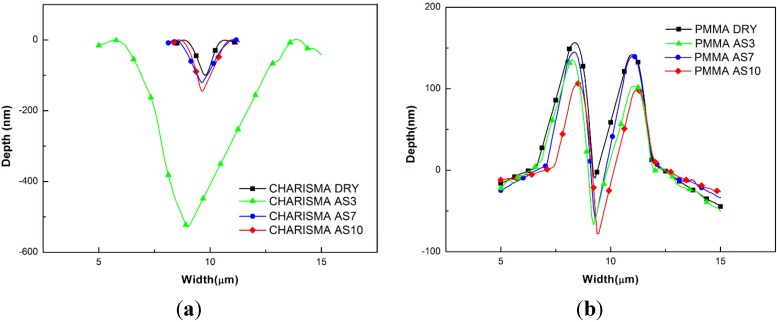

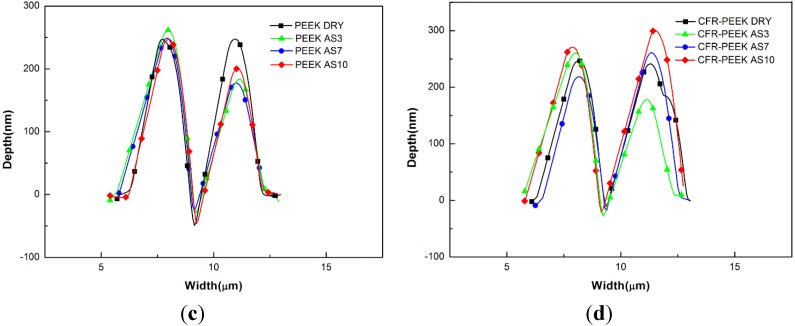
Residual scratch profiles of the (**a**) CHARISMA, (**b**) PMMA, (**c**) PEEK, and (**d**) CFR-PEEK.

The surface morphologies of the scratches of the samples under 1000 μN and the corresponding residual scratch profiles are displayed in Figure 7. The largest permanent set could be seen in the CHARISMA AS3 group, and the scratch depth was much deeper than that of other groups. For PEEK and CFR-PEEK, there were no statistical differences among treatment groups. The scratch depth and morphologies of all groups were similar. Although the morphologies of residual scratch profiles did not change, the scratch depth of PMMA became deeper after treatment.

Moreover, two different types of scratch deformations were observed in Figure 7. For CHARISMA, the scratch deformation mainly was beneath the surface. For PMMA, PEEK, and CFR-PEEK, the deformations were above the surface and heaped up along the scratch path. Because there were no permanent deformations in the carbon fiber region, the curves in Figure 7d were the residual scratch profiles of the CFR-PEEK matrix of different treatment groups.

## 5. Discussion

The environment of the oral cavity is a complex aqueous system. When a composite is used in the oral cavity, the dental composite absorbs water that diffuses internally through the resin matrix and flaws, most probably, through the filler-matrix interfaces. At the same time, the properties of the polymer are weakened by the release of unpolymerized monomer and deterioration of the network [24]. Moreover, polymer degradation does not occur as a result of isolated saliva permeation processes; multiple factors such as chewing, and thermal and chemical dietary changes may be responsible for biodegradation processes [25]. Along with the aging process, the capability of withstanding the deformation of polymer decreases, and may cause micro-leakage, secondary caries, filling dislodgement, and even catastrophic restorations fracture [26]. As a dental material, the degradation of PEEK and CFR-PEEK has not been investigated in the oral cavity. Therefore, this study tested and analyzed the nanoproperties of PEEK, CFR-PEEK, and their counterparts—which were treated with different pH artificial saliva—to evaluate whether PEEK and PEEK-based composites are a kind of suitable restoration materials in the future application.

### 5.1. The Reduced Elastic Moduli and Nanohardness

The present findings demonstrated that the reduced elastic moduli and nanohardness of the CHARISMA treatment group decreased, especially in of AS3 and AS10 groups, which are in accordance with a previously study [19]. The efficiency and durability of CHARISMA is mainly attributed to the intensity of the matrix network and silane coupling agent. In the process of saliva permeation, the interface between the filler particle and organic matrix has been considered as one of main solvent molecules diffusion pathways. This vulnerable path of the solvent molecules diffusion leads to the hydrolytic degradation of the silane-coupling agent and matrix network, and weakening of their reinforcing effect and surface properties [27]. In addition, the degradation of CHARISMA can be accelerated by different pH solutions, which is confirmed by the test value of AS3 group and AS10 groups. The influence of low pH on composite properties may be explained by the hydrolysis of ester groups present in the resin matrix. The alkaline reaction might adversely affect the water stability of the resin-to-filler bond at the organo-functional silane interface. The reaction in which siloxane bonds are attacked by hydroxyl ions leads to hydrolytic degradation [19].

After immersion in different pH value artificial saliva during the 30-day evaluation time interval, the results showed that the reduced elastic moduli and nanohardness of the PEEK matrix region of the treated specimens were quite similar to the respective values observed of in the control group specimen, expect that the reduced elastic moduli increased slightly. It indicates the structural stability of the PEEK at the nanoscale under the employed aging methods. These results are consistent with previous studies that found little or no influence of steam and gamma radiation sterilization processes on the nanomechanical properties of the PEEK matrix [2].

The finding of this study that the reduced elastic moduli and nanohardness of CFR-PEEK are improved by carbon fibers is consistent with a previous study [28]. Although the nanohardness and reduced elastic moduli increased due to the presence of reinforcing agents, the fiber-reinforced composite became a complex system containing various physical and chemical properties. It is reported that the overall mechanical performance of a heat-cured polymer composite, such as CFR-PEEK, is not only determined by the properties of different components, but also determined by the intensity and the durability of the interfacial area between the fibers and the polymer [29]. The interface between the matrix and the fibers, which is defined as the immediate chemical bond at the contact surface on the atomic scale in the process of cooling solidification [30]. The intensity of this interface is correlated to the degree of crystallinity and properties of PEEK matrix [31,32]. In the oral environment, the solvent molecules can be absorbed by a fiber-reinforced polymer composite in the ways of the diffusion or capillary action. Moreover, this process of absorption can be aggravated by the damage of bonding force and flaws in the interphase. The absorbed molecules can cause plasticization of matrix, differential swelling, embrittlement of macromolecular skeleton by hydrolysis and osmotic cracking [33]. For CFR-PEEK, once the solvent molecules penetrate into the polymer matrix, the molecule clusters are formatted between polymer chains. The different forms of degradation are affected by the location of these solvent molecules clusters. The two basic ways of absorption and diffusion of any solvent molecules are as follows: (1) molecules locate in the free space and slightly contact with the polymer chains, which leads to the increase in density. The dimensions of matrix will not change but the matrix will be plasticized. (2) The prior bonding between molecules and the polar sites of the polymer chain is formed. In this way, the diffused solvent molecules affect the intermolecular bonding between polymer chains, leading to the degradation of mechanical properties near the surface or throughout the bulk [34].

In this study, the reduced elastic moduli and nanohardness of the CFR-PEEK treatment group increased significantly. After the analysis of three different regions, the statistical results confirmed that both test values of the CFR-PEEK interphase increased, and the nanohardness of matrix increased slightly. Although Schambron *et al.* [35] reported no influence of the saline solution on the mechanical properties of fiber-reinforced PEEK in the macroscale, the nanomechanical properties of CFR-PEEK is influenced distinctly. According to the “free space way” of solvent molecules permeation, the obvious enhanced reduced elastic moduli and nanohardness may be attributed to the greater density caused by the water absorption and the dilatational pressure caused by the congregate molecules in the interface [34].

According to the past research, PMMA is a brittle material and is vulnerable when used in the oral cavity [36]. In this study, the treated samples of PMMA softened statistically because of the decreased in reduced elastic moduli and nanohardness. This result is consistent with the study of Assuncao *et al.* [37], who reported that storage in saliva could significantly reduce the hardness of the PMMA resin after 15, 30, and 60 days’ treatment. Moreover, it is reported that after 24 h immersion in the water, the PMMA matrix can significantly swell in the lateral and vertical directions [38], indicating that the solvent molecules penetrate into the PMMA matrix and break the intermolecular bonding between polymer networks. Once the matrix network is broken, the properties of PMMA will get worse inevitably.

### 5.2. The Nanoindentation Viscoelasticity

In this study, all the materials were plasticized after immersion treatment. The higher plasticity index and lower percentage of nanoindentation recovery indicate that more permanent deformation occurs in one indentation. Moreover, high creep deformation and low recovery of polymer exhibit poor resistance to mechanical stress and thus may influence the long-term clinical durability of their restorations [39].

Although the plasticity index increased, the percentage of nanoindentation recovery of CHARISMA AS3 and CHARISMA AS10 groups increased significantly. In the CHARISMA AS10 group, it is possible that due to the destruction of the connection between filler and matrix, the higher elastic recovery is mainly performed by the resin matrix. Furthermore, in the CHARISMA AS3 group, the network of resin matrix might be degraded by the hydrolysis of lactic acid with the sharply increased nanoindentation creep.

For the nanoindentation creep of PEEK, no significant difference was observed between the control group and experimental groups, indicating that the PEEK has more stable resistance to a long-time mechanical stress compared with other materials. However, the slightly increased plasticity index and the decreased percentage of nanoindentation recovery indicate that the capability of the elastic recovery of PEEK is weakened. These results are consistent with those observed by Iqbal *et al.* [40]; they also reported the slight plasticization of the PEEK matrix after water storage. For PEEK, the almost unchanged plasticity index proves that this polymer has great durability for the employed aging treatment.

**Figure 8:**
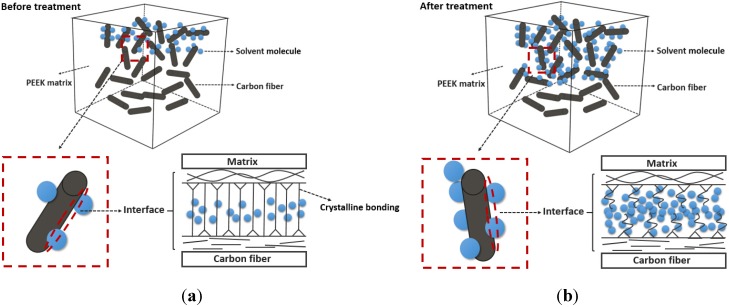
Schematic of solvent molecules penetrating ways of the CFR-PEEK interface. (**a**) Before AS3 and AS10 treatment, the intensity of the interface between the matrix and fiber was not weakened, and the solvent molecules were limited in number; (**b**) Once the crystalline bonding of the interface was broken by the AS3 and AS10 treatment, more molecules flowed into the interface and plasticized the matrix further.

After the storage treatment of artificial saliva, in CFR-PEEK AS3, CFR-PEEK AS7, and CFR-PEEK AS10 groups, the plasticity index increased and the percentage of nanoindentation recovery decreased significantly. These results indicate that the storage treatment leads to the plasticization of the materials and the solvent molecules of artificial saliva penetrate into the matrix in the “free space way” [34]. Compared to PEEK, the obvious plasticization of the treated CFR-PEEK may be associated with the damage of bonding force in the interface between polymer matrix and carbon fiber [41]. Along with the weakened intensity of the interface, the contact area of artificial saliva increases and more solvent molecules will penetrate into the interface. Moreover, the increased trends of plasticization properties of CFR-PEEK AS3 and CFR-PEEK AS10 groups seem the same as in the CFR-PEEK AS7 group, but to a further degree. In these groups, the degree of crystalline bonding in the interface may be weakened by the acid and alkali condition, allowing more molecules fill the free space and plasticize the matrix. (see Figure 8).

In consideration of the plasticity index and percentage of nanoindentation recovery, the PMMA was plasticized by the different pH value artificial saliva. The exacerbated degradation in PMMA AS3 and PMMA AS10 groups may be aggravated by the acid and alkali condition. Because the formation of a hydroxyl species can cause the chain scission of PMMA [42], the further destruction of the polymer matrix may contribute to the synergistic effect of artificial saliva and sodium hydroxide in the PMMA AS10 group. Nevertheless, the enhanced destructive effect of the PMMA AS3 group depends whether on the damage of intermolecular bonding between networks or on the scission of polymer chains need further investigation.

### 5.3. The Nanoscratch Property

After treatment (see Figure 6), the friction coefficient of CHARISMA increased and the residual scratch profiles became deeper, especially in the CHARISMA AS3 group. With higher friction coefficient and deeper residual scratch profiles, it is consistent with the aforementioned presumption that the network of resin-based composite CHARISMA was damaged after storage in the AS3 solution.

The friction coefficient and the scratching profiles of the PEEK matrix can be seen in Figure 6 and Figure 7. It can be seen that the surfaces of CFR-PEEK and PEEK are stable under different pH value solutions, and the friction coefficient is almost invariant, indicating that the employed aging treatment has basically no effects on their abrasion resistance.

Taking the increased friction coefficient and the deeper nanoscratch depth into consideration, it can be concluded that the abrasion resistance of PMMA is weakened by the treatment. According to a previous study and aforementioned ways of permeation [34], it can be presumed that solvent molecules permeate into this kind of PMMA in the “polar site bonding way”, and the intensity of matrix network is destroyed.

In this study, all the results prove that performance of unfilled PEEK is steady under the employed aging treatment. It can be deduced from the test data of CFR-PEEK that the PEEK matrix with filler is less stable than that without filler in the nanoscale. The durability of PEEK-based composites may be mainly depended on the stability of the interface between matrix and fillers. Compare to PEEK, PMMA and CHARISMA are more susceptible to the influence of different pH artificial saliva treatment. With these outstanding properties, it can predict that PEEK has excellent potential for future dental application.

Within the limitations of this study, only the microscopic aspects of mechanical properties were investigated. The macroscopic mechanical performance and the changes of chemical properties of PEEK and CFR-PEEK under various simulated oral conditions need further investigation in the future research.

## 6. Conclusions

The nanomechanical properties of PEEK were not affected by immersion in different pH artificial saliva at physiological temperatures over 30 days. The reduced elastic moduli, nanohardness, viscoelasticity, and friction performance remained unaffected by the absorption of solution. The simulative oral liquid environment caused no significant changes of the PEEK.

Once the PEEK reinforced by the filler, the nanomechanical properties of CFR-PEEK were enhanced. However, the stability of nanomechanic properties of CFR-PEEK became worse after the employed aging treatment.

Compared to PEEK, CHARISMA exhibited more degradation and was less stable after the storage treatment. The acidic artificial saliva had more destructive effects on CHARISMA.
